# Dichotic spectral integration range for consonant recognition in listeners with normal hearing

**DOI:** 10.3389/fpsyg.2022.1009463

**Published:** 2022-10-20

**Authors:** Yang-Soo Yoon, Dani Morgan

**Affiliations:** Laboratory of Translational Auditory Research, Department of Communication Sciences and Disorders, Baylor University, Waco, TX, United States

**Keywords:** dichotic hearing, spectral integration, binaural integration, consonant recognition, articulation-index gram

## Abstract

Dichotic spectral integration range, or DSIR, was measured for consonant recognition with normal-hearing listeners. DSIR is defined as a frequency range needed from 0 to 8,000 Hz band in one ear for consonant recognition when low-frequency information of the same consonant was presented to the opposite ear. DSIR was measured under the three signal processing conditions: (1) unprocessed, (2) target: intensified target spectro-temporal regions by 6 dB responsible for consonant recognition, and (3) target minus conflicting: intensified target regions minus spectro-temporal regions that increase confusion. Each consonant was low-pass filtered with a cutoff frequency of 250, 500, 750, and 1,000 Hz, and then was presented in the left ear or low-frequency (LF) ear. To create dichotic listening, the same consonant was simultaneously presented to the right ear or high-frequency (HF) ear. This was high-pass filtered with an initial cutoff frequency of 7,000 Hz, which was adjusted using an adaptive procedure to find the maximum high-pass cutoff for 99.99% correct consonant recognition. Mean DSIRs spanned from 3,198–8,000 Hz to 4,668–8,000 Hz (i.e., mid-to-high frequencies were unnecessary), depending on low-frequency information in the LF ear. DSIRs narrowed (i.e., required less frequency information) with increasing low-frequency information in the LF ear. However, the mean DSIRs were not significantly affected by the signal processing except at the low-pass cutoff frequency of 250 Hz. The individual consonant analyses revealed that /ta/, /da/, /sa/, and /za/ required the smallest DSIR, while /ka/, /ga/, /fa/, and /va/ required the largest DSIRs. DSIRs also narrowed with increasing low-frequency information for the two signal processing conditions except for 250 vs. 1,000 Hz under the target-conflicting condition. The results suggest that consonant recognition is possible with large amounts of spectral information missing if complementary spectral information is integrated across ears. DSIR is consonant-specific and relatively consistent, regardless of signal processing. The results will help determine the minimum spectral range needed in one ear for consonant recognition if limited low spectral information is available in the opposite ear.

## Introduction

Normal hearing (NH) listeners receive the same or similar auditory input from each ear, and the input is then sent to the higher auditory system for further processing, such as spectral integration (Ronan et al., 2004; Hall et al., 2008; Fox et al., 2011; Räsänen and Laine, 2013; Grose et al., 2016). However, individuals with hearing loss may receive different spectral information from each ear and are forced to integrate them across the ears, that is, dichotic spectral integration (Tononi, 2010; Kong and Braida, 2011; Yang and Zeng, 2013; Reiss et al., 2014; Obuchi et al., 2015). This dichotic spectral integration occurs when different frequency information is dichotically and simultaneously presented to both ears. The improvement in the performance of listeners with hearing loss as signal bandwidth widens is thought to reflect the ability of the auditory system to integrate information across a wide frequency range in complex sounds (Spehar et al., 2008; Happel et al., 2010). Regardless of hearing status, dichotic spectral integration is important for efficient communication, such as speech perception. However, it is hard to find dichotic spectral integration studies with NH and hearing-impaired listeners. In the present study, a frequency range needed in the right ear for consonant recognition was determined when low-frequency information of the same consonant presented to the right ear is presented in the left ear in NH listeners. In this study, this frequency range was named a “dichotic spectral integration range (DSIR).”

It is known that consonant recognition requires the listener’s ability to discriminate specific spectral and temporal acoustic cues such as voicing, the onset of the noise burst, and spectral and temporal transitions (Miller and Nicely, 1955; Stevens and Klatt, 1974; Stevens and Blumstein, 1978; Blumstein and Stevens, 1979, 1980). In contrast, a few studies measured the range of spectral information needed for consonant recognition. Lippmann (1996) measured consonant–vowel–consonant syllable recognition in NH listeners when low-frequency information below 800 Hz was combined with high-frequency information above 4,000 Hz in a monotic listening condition (i.e., different frequency information is simultaneously presented to the same ear). This monotic spectral integration study showed that removing midfrequency consonant information (800–4,000 Hz) did not significantly alter consonant recognition. Ronan et al. (2004) did not determine the spectral integration range but demonstrated a relationship between speech perception and monotic spectral integration in NH listeners. They filtered consonants in two widely separated bands (0–2,100 Hz and 2,100–4,500 Hz) of speech. They observed that consonant enhancement is related to the ability of integrating widely separated two bands.

Some other studies showed that dichotic spectral integration (i.e., different frequency information is dichotically and simultaneously presented to both ears.) facilitates sentence perception (Hall et al., 2008; Grose et al., 2016). Hall and colleagues first determined the bandwidths required for approximately 15–25% correct sentence recognition in quiet and noise conditions in listeners with NH and hearing loss (Hall et al., 2008). They then adaptively varied the bandwidth of filtered sentences centered on low (500 Hz) and high (2,500 Hz) frequencies and measured speech perception when the two bandwidths were presented simultaneously to both ears. NH and hearing-impaired listeners observed higher percent performance (64–94%) with dichotic spectral listening compared to a 30–50% additive combination of information presented in the single-band conditions. Grose et al. (2016) also reported similar results as Hall et al. (2008) study but with middle-aged and older NH listeners.

The ability to integrate spectral information across ears may be affected when useful frequency information for speech perception is manipulated, such as being intensified or eliminated. Allen’s group identified specific frequency and time regions for the consonant perception that resulted in an improved consonant recognition, called “target frequency and time regions.” They also identified specific frequency and time regions that lead to significant consonant confusions, called “conflicting frequency and time regions” (Li et al., 2010, 2012). Consonant recognition was measured with + 6 dB gain on the target (frequency and time) regions and complete removal of the conflicting (frequency and time) regions for consonants. The results from these four studies indicated that the intensified target and removal of the conflicting regions enhance consonant recognition by a minimum of 3 percentage points to a maximum of 70 percentage points. This type of signal processing with the target and conflicting regions will enhance speech perception in listeners with normal hearing. However, listeners with hearing loss with or without devices may not integrate these regions appropriately across ears due to abnormal binaural spectral integration, i.e., fusion and averaging of information from widely different frequency regions (Reiss et al., 2014). This can lead to interference, as was shown for vowel integration (Fowler et al., 2016). Under this listening condition, some listeners may experience spectral interference rather than spectral integration. For example, Fowler et al. (2016) demonstrated that bimodal patients who had better residual hearing (< 60 dB HL at 250 and 500 Hz) in the hearing aid ear received improved speech perception in quiet when low-to-mid (approximately 440–982 Hz) frequencies in cochlear implant ear were removed. Removing mid-frequency information processed by cochlear implant ear may reduce bimodal interference and/or enhance bimodal integration. It is also possible that the AI-Gram signal processing would result in ear-dominance listening when the target and conflicting regions are processed by one ear with a better performing ear (e.g., cochlear implant ear in bimodal hearing). An ear dominance listening results in information presented to one ear being primarily processed and perceived, while information presented to the opposite ear is less utilized and perceived (Reiss et al., 2016). Under ear-dominance listening, dichotic spectral integration will be less affected with the poorer ear (i.e., hearing aid ear in bimodal hearing). So, the findings of Li et al. studies (Li et al., 2010, 2012) led to the working hypothesis that the DSIR will be significantly reduced if target regions are intensified while the conflicting regions are removed. It is also hypothesized that DSIRs will be narrowed with increasing low-frequency information in the opposite ear.

In summary, previous studies demonstrate that spectral integration within and between ears is important for speech perception using two broad frequency bands (Ronan et al., 2004; Hall et al., 2008; Räsänen and Laine, 2013; Grose et al., 2016). However, spectral integration may occur at specific narrower frequency bands, and additional spectral integration on other bands may not be critical for speech perception. It is also possible that the spectral integration range is listener specific for speech recognition. For example, individuals with different degrees of hearing loss in one ear may need different ranges of spectral information in the opposite ear for good speech recognition. Another challenging aspect of the previous studies is the use of sentences (Hall et al., 2008; Grose et al., 2016). Sentences are more realistic stimuli compared to tones or nonsense syllables. However, the minimum spectral ranges required for sentence perception would be similar regardless of the use of different sentences. Measuring DSIRs for phonemes (i.e., basic units of sentences) will provide us discrete information which can be effectively used in training machine-learning algorithms. In the present study, DSIRs were determined for individual consonant recognition in the right ear when different amounts of low-frequency information were presented to the left ear in NH listeners. The DSIR measurement was administered under the three signal processing conditions: unprocessed, with target frequency and time regions intensified by +6 dB gain (i.e., target condition), and both the target regions intensified, and conflicting regions removed (i.e., target minus conflicting or target-conflicting condition). The results of the present study will help determine the minimum spectral range needed in one ear for individual consonant recognition if limited low spectral information is available in the opposite ear. The results can also serve as control data for future studies with hearing-impaired listeners and bimodal users.

## Materials and methods

### Subjects

Fourteen NH adults participated (11 females and three males; average age: 24 ± 6.7). A reason for this imbalance of subject gender was that the subjects were mainly recruited from Robbins College of Health and Human Sciences at Baylor University, where female students outnumber male students. All subjects were native speakers of American English. All participants had thresholds better than 20 dB HL (hearing level) for both ears at audiometric frequencies ranging from 250 to 8,000 Hz. Interaural threshold differences were less than 10 dB HL. All procedures were approved by the Baylor University Institutional Review Board (#1253711). The Board has determined that the research agrees with the declaration of Helsinki.

### Stimuli

Stimuli included 14 frequently used American English consonants with the common vowel /ɑ/ (/pa/, /ba/, /ta/, /da/, /ka/, /ga/, /ma/, /na/, /fa/, /va/, /sa/, /za/, /ʃa/, and /ʃa/; Hayden, 1950). Each consonant was produced with a sampling frequency of 44,100 Hz by a single female talker whose average fundamental frequency was 228 Hz. Completely silent parts from both onsets and offsets of consonant syllables were identified on time waveforms and spectrograms and manually removed. The average duration and standard deviation (SD) of consonants was 406.57 ± 102.61 ms. The duration of each consonant is provided in Table 1. To limit the spectral range of consonants to 0–8,000 Hz, each consonant was low-pass filtered with a cutoff frequency of 8,000 Hz (IIR second-order Butterworth with 12 dB/oct roll-off and a zero-phase shift). All stimuli were then normalized to have the same long-term root-mean-square energy (65 dBA sound pressure level or SPL). The stimuli was delivered to both ears *via* circumaural headphones (Sennheiser HDA-200) at the subject’s most comfortable level (ranges: 50–70 dB SPL), which was established by the subjects’ responses to the 14 unprocessed consonants from the stimuli listed above in quiet according to the Cox loudness rating scale (Cox, 2005).

**Table 1:** The target and conflicting frequency and time regions used for the AI-Gram processing (Yoon, 2021).

Consonants	Duration [ms]	Target frequency [kHz]	Conflicting frequency [kHz]	Target time [ms]
/pa/	240	0.3–7.4	1.4–2.0	32–62
/ba/	331	0.3–4.5	0.6–2.2	7–22
/ta/	338	3.0–7.4	1.6–2.8	42–62
/da/	240	4.0–7.8	1.4–2.8	38–48
/ka/	447	1.4–2.0	5.0–7.8	30–50
/ga/	348	1.4–2.0	3.9–5.0	10–30
/ma/	350	0.5–1.3	1.2–1.9	25–55
/na/	400	1.5–2.2	0.4–0.9	77–127
/fa/	548	0.6–2.2	3.0–7.8	141–166
/va/	349	0.6–1.4	1.4–4.4	16–46
/sa/	501	3.9–7.8	5.4–7.8	80–115
/za/	501	3.6–7.8	3.5–5.4	90–120
/ʃa/	549	2.0–3.7	4.0–7.8	40–160
/ʒa/	550	1.9–3.7	5.4–7.8	15–115

Duration of each consonant is also given. The entire range of frequency that was used for the identification of the target frequency regions was 0–8 kHz.

### Articulation index-gram processing on the target and conflicting frequency and time regions

**Figure 1:**
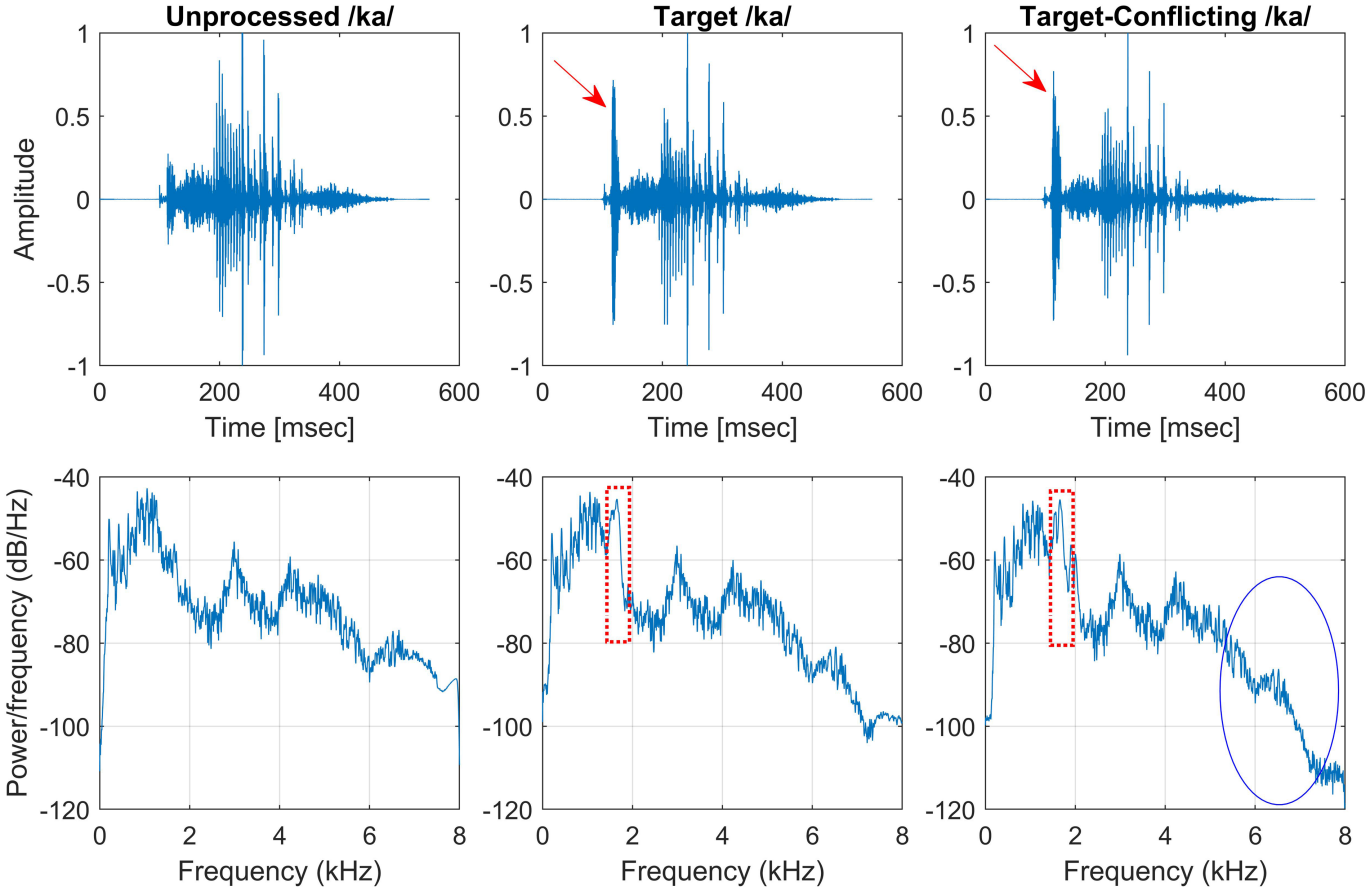
Stimulus wave forms (top panels) and power spectra (bottom panels) for the unprocessed (left column), target (middle column), and target-conflicting (right column) conditions forn/ka/. Arrows indicate the target portion of stimulus wave forms. Dotted rectangles and solid oval on the power spectra indicate the target and conflicting regions, respectively.

The same target frequency and time regions of consonants used in our previous study in NH listeners (Yoon, 2021) was employed in this study. Figure 1 shows timewave forms and power spectrum for the unprocessed, target, and target-conflicting processed /ka/. Arrows indicate the target portion of timewave forms. Dotted rectangles and solid ovals on the power spectrums indicate the target and conflicting regions, respectively. The AI-Gram was originally developed by Li et al. (2010, 2012). The AI-Gram was implemented on the MATLAB platform (The MathWorks, 2017) for our conditions (Yoon, 2021). The AI-Gram construction procedures are explained in detail in Yoon (2021). A full discussion of how consistent the target and conflicting frequency regions are with respect to earlier studies (Li et al., 2010, 2012) can also be found in Yoon (2021).

In brief, using a low-pass and high-pass filtering scheme (IIR second-order Butterworth with −12 dB/oct roll-off and a zero-phase shift for both filters), the target frequency regions were identified. These target regions for each consonant are the frequency regions responsible for significant changes in consonant recognition. For example, /ka/ was presented and its perception scores were considerably improved (from 40 to 90%) when the low-pass filter (LPF) cutoff was moved from 1.4 to 1.5 kHz. So, the lower edge of the target frequency would be 1.4 kHz. When the high-pass filter (HPF) cutoff was moved from 2.0 to 2.1 kHz, the recognition of /ka/ considerably dropped (from 90 to 40%). So, the upper edge of the target frequency would be 2.0 kHz. Therefore, the final target frequency region would be 1.4–2.0 kHz for /ka/. This target frequency region included the spectral region (i.e., 1.4 kHz) that leads to improved consonant perception when LPF cutoff frequency raised from 1.4 kHz to 1.5 kHz but excluded the spectral region (i.e., 2.1 kHz) that leads to a potential deteriorated consonant perception when HPF cutoff frequencies raised from 2.0 to 2.1 kHz. The conflicting frequency regions are the frequency regions that yielded the peak errors of the most confused consonants and 20% less than the peak error from both filtering schemes. For example, when /ʃa/ was presented, the recognition of the confused consonant /sa/ reached 24% when the LPF cutoff was 4.0 kHz and a maximum of 30% when the cutoff was moved from 4.0 to 4.1 kHz (i.e., 24% is 20% below the peak 30% error). Therefore, the lower edge of the conflicting frequency would be 4.0 kHz. When the HPF cutoff was 7.8 kHz, the recognition of the confused consonant /sa/ reached a score of 24% and a maximum of 30% when the cutoff was moved from 7.8 to 7.7 kHz. So, the upper edge of the conflicting frequency would be 7.8 kHz. Thus, the final conflicting frequency range would be 4.0–7.8 kHz for recognition of the consonant /ʃa/. Full descriptions of selection criteria for target and conflicting regions can be found in Yoon (2021).

Analogously, using a truncation approach, the target time regions for each consonant was identified by finding the time segment of the consonant responsible for significant change in consonant recognition. The initial duration of each consonant was 3% of the total duration from the onset (i.e., the remaining 97% of the consonant was truncated out) so that minimal consonant information was presented. The duration of the consonant was increased by 1 ms when a participant’s response was incorrect. If perception scores for */*ka*/* dropped significantly (i.e., from 90 to 40%) when the time-truncation point increased from 30 to 50 ms the onset of the consonant, it suggests that important temporal cues resided within the 30–50 ms time window. Again, these target frequency and time regions used for the current study were obtained from NH listeners in the binaural hearing condition and in quiet (Yoon, 2021). After identifying the target frequency and time regions for each of the 14 consonants using the AI-Gram, a 6-dB gain was applied to those target frequency and time regions for each consonant (i.e., other frequency and time regions for each consonant were intact). The conflicting frequency and time regions were also removed. For the three consonants (/pa/, /ba/, and /za/) with overlapping target and conflicting frequency ranges (Figure 2 in Yoon, 2021), the target frequency ranges were intensified, while the overlapped conflicting frequency ranges were not removed. It should be noted that AI-Gram does not have the ability to apply a 6 dB gain and removal on the exact target and conflicting regions. So, some variations should be expected on the power spectrums for the target and target-conflicting processing conditions, as shown in Figure 1. The completed AI-Gram processing was then verified by five adult NH listeners. The verification procedures can also be found in Yoon (2021). Table 1 lists the resultant target and conflicting frequency and time regions. Note that the target time region in Table 1 indicates a temporal duration of consonants from the onset of the consonant.

**Figure 2:**
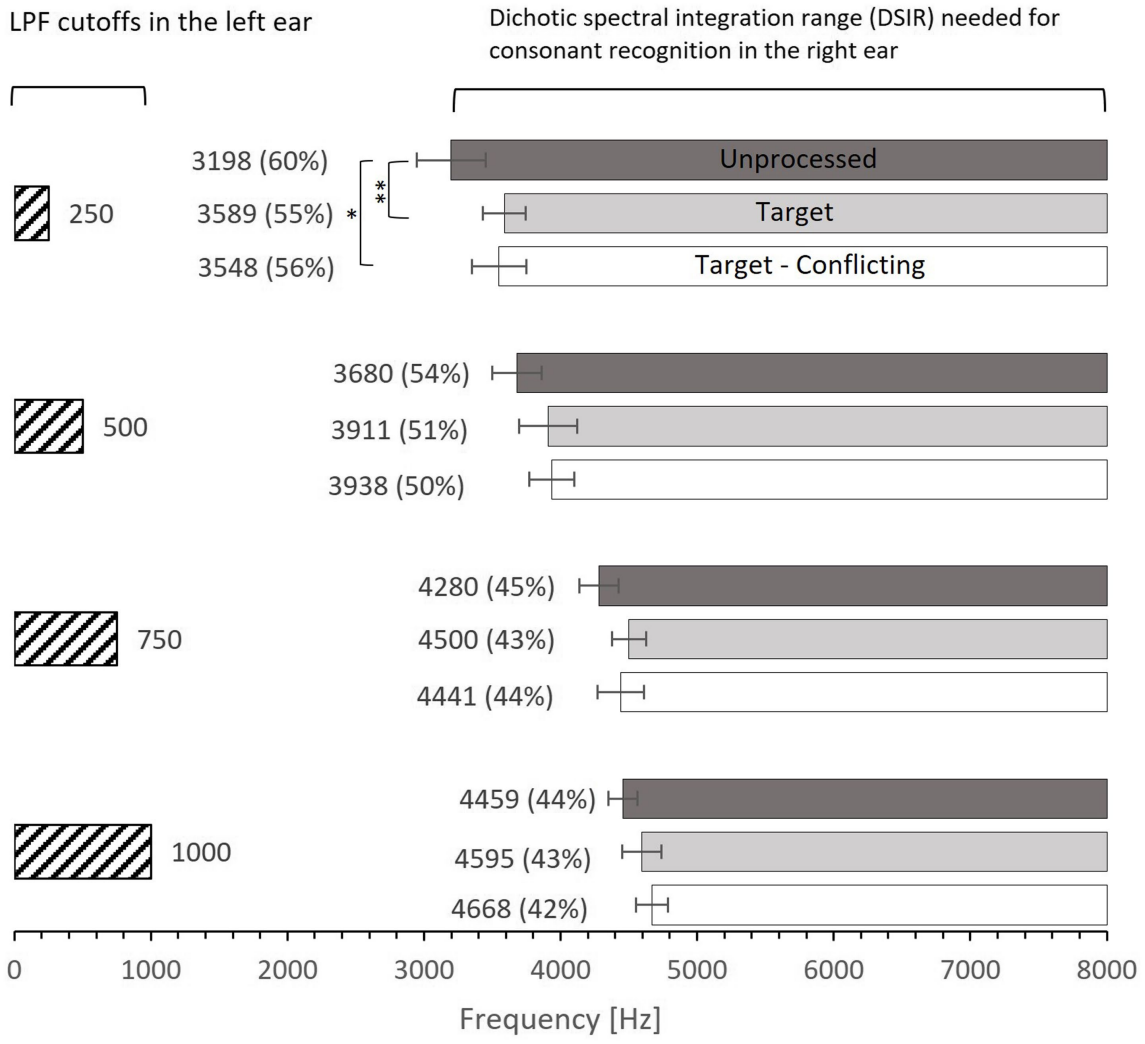
Mean dichotic spectral integration range (DSIR) needed for consonant recognition in the HF ear for each LPF cutoff frequency in the LF ear. Dark- and light-filled bars indicate the unprocessed and target conditions, while the open bars indicate the target-conflicting condition. Numbers in the parentheses are the percentages of DSIR out of 0–8,000 Hz band required for consonant recognition in the HF ear (e.g., 60% for the unprocessed condition at 250 Hz was obtained from 8,000–3,198 Hz = 4,802 Hz, which is 60% of the 0–8,000 band). Error bars indicate standard errors. ^**^p < 0.01 and ^*^p < 0.05.

### Procedure

The DSIR was binaurally measured in quiet under three signal processing conditions: (1) unprocessed, (2) target: intensified target frequency and time regions responsible for consonant recognition, and (3) target-conflicting: combined intensified target frequency and time regions and removed conflicting frequency and time regions responsible for consonant confusions. Subjects were seated in a single-walled sound-treated booth (Industrial Acoustics Company). Before formal testing, a 30-min familiarization on all 14 consonants was binaurally provided for the target and target-conflicting signal processing conditions in a quiet environment (15-min each). Each consonant was low-pass filtered (IIR fifth-order Butterworth with 30 dB/oct roll-off) in the left ear, with one of the four fixed cutoff frequencies: 250, 500, 750, and 1,000 Hz. Group delay created by filtering was removed by applying zero-phase filtering technique. These cutoff frequencies were purposefully chosen because they are the typical frequencies of residual hearing in individuals who utilize bimodal hearing (Smith-Olinde et al., 2004; Jürgens et al., 2011; Bianchi et al., 2016; Patel and McKinnon, 2018; Varnet et al., 2019; Yoho and Bosen, 2019). Results from these chosen cutoff frequencies can be used for future comparison with data that will be measured in individuals with hearing aids and cochlear implants. In the right ear, the same consonant was presented with an initial HPF cutoff frequency of 7,000 Hz (IIR fifth-order Butterworth with 30 dB/oct roll-off). Zero group delay was achieved by applying a zero-phase filtering on filtered signals. An incorrect response lowered the cutoff frequency in 100-Hz decrements (i.e., the cutoff frequency was reduced from 7,000 to 6,900 Hz). So, low-frequency information was presented to the left ear which was designated as the “low-frequency or LF ear,” and the high-frequency information was presented to the right ear which was designated as the “high-frequency or HF ear.” Under these LF and HF ear settings, the stimulus was dichotically and simultaneously delivered *via* an audiometer (GSI AudioStar Pro) to Sennheiser HDA-200 circumaural headphones. In fixed block trials, DSIR was determined, using the 15-alternative forced-choice paradigm, along with the additional option of “none of these.” With each of the four fixed low-pass filter cutoff frequencies used in the LF ear, each consonant was presented five times for each signal processing, and the order of consonant presentation was fully randomized. The DSIR was determined when the consonant presented was correctly selected three times in a row. These procedures were repeated for the unprocessed, target, and target-conflicting signal processing conditions. No trial-by-trial feedback was provided during the test. The complete test protocol (3 signal processing conditions × 4 LPF cutoff frequencies × 14 consonants × 5 repetitions), including five-minute breaks (at least two breaks per hour and instructed to take breaks as needed) and the consenting process, took approximately 11 h per listener, requiring four separate visits.

### Data analysis

**Figure 3:**
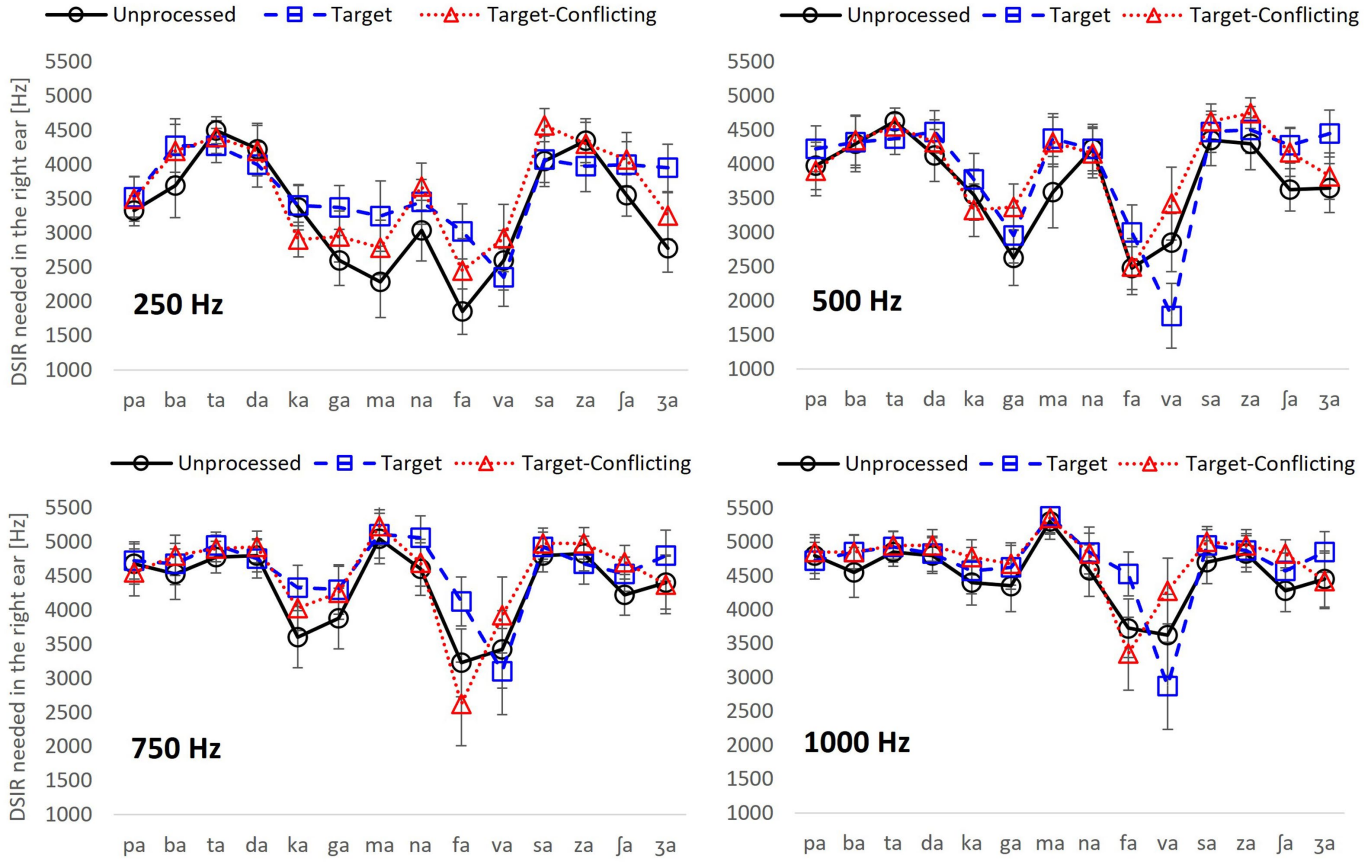
Dichotic spectral integration range (DSIR) out of 0–8,000 Hz band in the HF ear with the standard errors for individual consonant as a function of signal processing for each LPF cutoff frequency in the LF ear.

**Figure 4:**
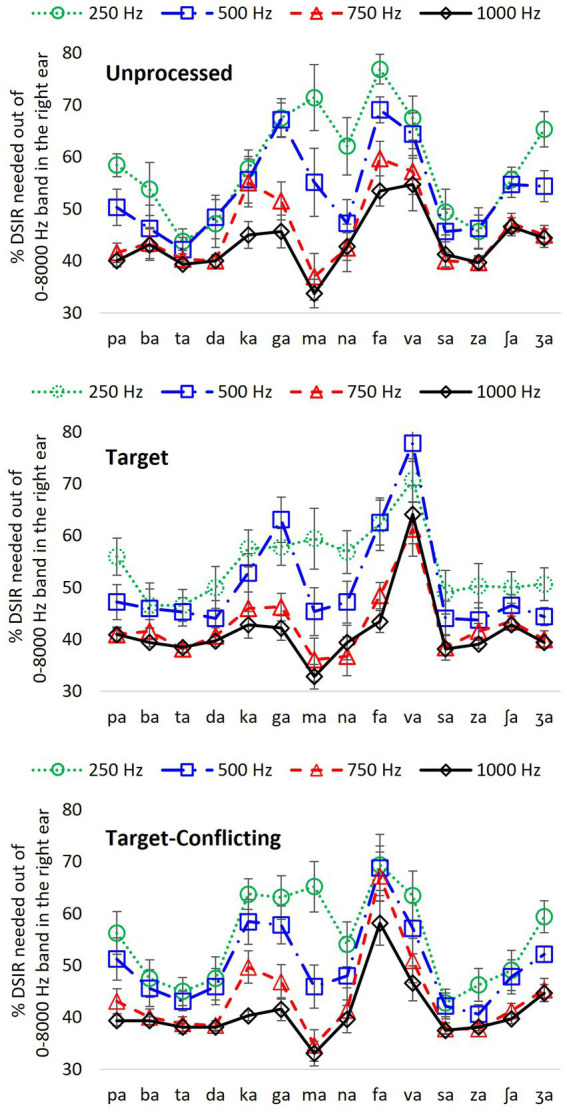
Percentage of DSIR out of 0–8,000 Hz band in the HF ear for individual consonant recognition as a function of LPF cutoff frequency in the LF ear for each signal processing condition.

**Figure 5:**
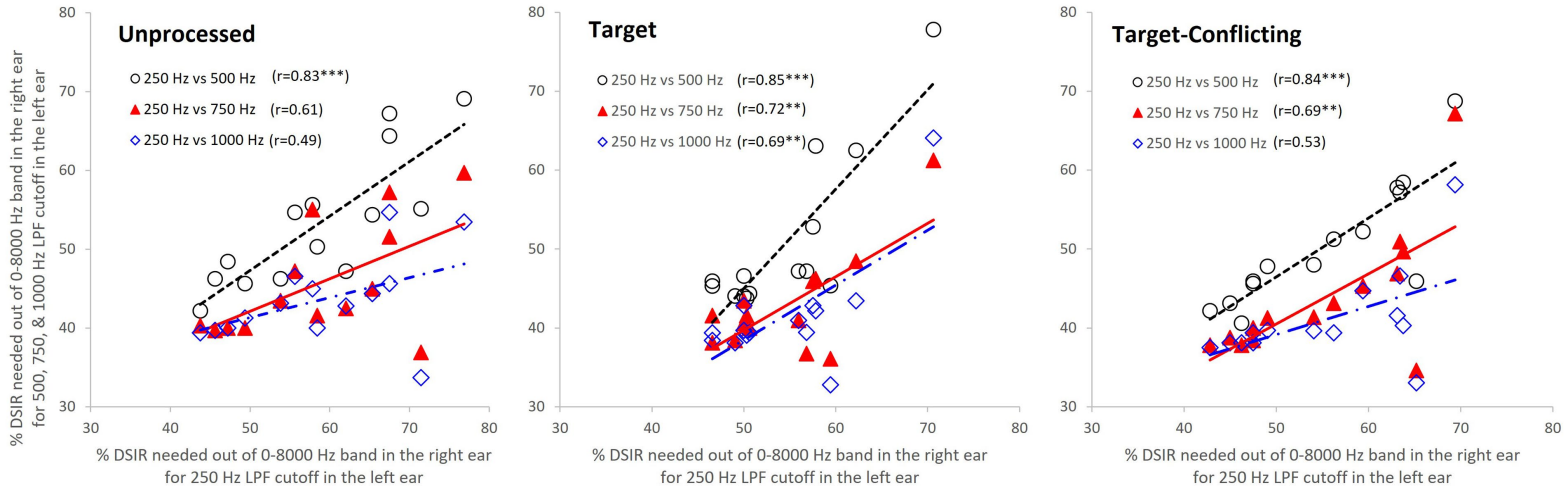
Scatter plots among percentages of DSIRs. Each data point represents the average DSIR percentage of each syllable across subjects. The *X*-axis indicates DSIRs for 250 Hz LPF cutoff frequency, while the y-axis indicates DSIRs for LPF cutoff frequencies of 500, 750, and 1,000 Hz. The open circle is a scatter plot of DSIRs of 250 and 500 Hz LPF cutoff frequencies, while the filled triangle is a scatter plot of DSIRs of 250 and 750 Hz LPF cutoff frequencies. The open diamond is a scatter plot of DSIRs of 250 and 1,000 Hz cutoff frequencies. ^***^p < 0.001 and ^**^p < 0.01.

Parametric statistics were used with Sigma Plot (SYSTAT, 2021). Before performing statistical analyses, the normality (Shapiro–Wilk) test and equal variance (Brown-Forsythe) test were performed, and all passed. To determine the main effect of the AI-Gram signal processing and LPF cutoff frequencies on mean DSIRs (Figure 2), a two-way repeated measures analysis of variance (ANOVA) was performed with two within-subject factors: the AI-Gram (Unprocessed, Target, and Target-Conflicting) and LPF cutoff frequency (250, 500, 750, and 1,000 Hz). A two-way repeated ANOVA was also performed with two within-subject factors (i.e., the AI-Gram and each consonant) to determine how DSIR for individual consonants was affected by the AI-Gram signal processing (Figure 3). A two-way repeated ANOVA was performed with two within factors (LPF cutoff frequency and each consonant) to determine how the DSIR of each consonant was affected by the LPF cutoff frequency used in the LF ear (Figure 4). Pearson correlation analyses were conducted to determine any systematic relationship in the DSIRs with different LPF cutoff frequencies (Figure 5). The results of all statistical analyses were assessed against an alpha level of 0.05 with a two-tailed test. Planned multiple comparisons were performed using an overall alpha level of 0.05 with the Bonferroni correction.

## Results

### Mean DSIR

Figure 2 shows mean DSIR with the standard error for each LPF cutoff frequency used for the LF ear. All DSIRs should be interpreted as lower bound frequencies required for consonant recognition from the 0–8,000 Hz band. For example, DSIR of 3,198 Hz (for 250 Hz cutoff frequency and the unprocessed conditions) means that a frequency range of 3,198–8,000 Hz was required for consonant recognition in the HF ear when low-frequency information below 250 Hz was presented to the LF ear. The numbers in parentheses are the percentages of DSIRs needed for consonant recognition from the 0–8,000 Hz band. For instance, 60% (for 250 Hz cutoff frequency and the unprocessed conditions) means that the DSIR of the 3,198–8,000 Hz covers 60% of the upper portion of the 0–8,000 Hz band. The results show that consonant recognition was achieved with large amounts of spectral information missing. DSIRs narrowed (i.e., required less spectral information) with increasing the LPF cutoff frequency. A two-way repeated measure analysis of variance (ANOVA) showed a significant effect of AI-Gram processing effect, *F*(2,78) = 4.28, *p* = 0.02 and of the LPF cutoff frequency on DSIRs, *F*(3,36) = 46.55, *p* < 0.001. However, no significant interactions were observed between the signal processing and the LPF cutoff frequency, *F*(6,78) = 1.29, *p* = 0.32. All pairwise multiple comparisons across signal processing conditions, with Bonferroni correction for the AI-Gram processing, showed that only two pairs were significant within the cutoff frequency of 250 Hz: unprocessed vs. target (*p* = 0.005) and unprocessed vs. target-conflicting (*p* = 0.01), indicated by asterisks in Figure 2. Across the LPF cutoff frequency, differences between all pairs are significant except for pair 750 Hz vs. 1,000 Hz within all three signal-processing conditions and pair 250 vs. 500 Hz within the target condition. Details are given in Table 2.

**Table 2:** Pairwise multiple comparisons for LPF cutoff frequencies in the LF ear.

	250 vs. 500 Hz	250 vs. 750 Hz	250 vs. 1,000 Hz	500 vs. 750 Hz	500 vs. 1,000 Hz	750 vs. 1,000 Hz
Within unprocessed	^**^	^***^	^***^	^***^	^***^	ns
Within target	ns	^***^	^***^	^***^	^***^	ns
Within target-conflicting	^*^	^***^	^***^	^***^	^***^	ns

*** *p* < 0.001;

** *p* < 0.01;

* *p* < 0.05.

ns stands for not significant.

### Dichotic spectral integration range for individual consonant

To determine what frequency range is needed for the recognition of each consonant, DSIR per consonant was plotted as a function of the signal processing condition for each LPF cutoff frequency in Figure 3. Two overall findings are that DSIRs are highly consonant-specific, and the patterns of DSIRs are similar between 250 and 500 Hz LPF cutoff frequencies, as well as between 750 and 1,000 Hz LPF cutoff frequencies.

For the LPF cutoff frequency of 250 Hz, a two-way repeated measure ANOVA showed that DSIRs were significantly different across consonants, *F*(13,338) = 10.70, *p* < 0.001 but not across the AI-Gram signal processing, *F*(2,338) = 1.91, *p* = 0.17. Significant interactions were observed, *F*(26, 338) = 1.82, *p* = 0.009. Based on the shapes of the DSIRs, there were two subgroups: five consonants (/ka/, /ga/, /ma/, /fa/, and /va/), requiring wide DSIRs, and the remaining nine consonants requiring relatively narrow DSIRs. This subgrouping was supported by the results of pairwise multiple comparisons with Bonferroni correction (Table 3). These five consonants required significantly wider DSIRs compared to the other nine consonants. For the LPF cutoff frequency of 500 Hz, significant difference in DSIRs were observed across consonants, *F*(13,338) = 14.36, *p* < 0.001, but no significant effect of the AI-Gram signal processing, *F*(2,338) = 0.94, *p* = 0.40. Significant interactions were observed, *F*(26, 338) = 2.52, *p* < 0.001. Observed with the 250 Hz, the same five consonants (/ka/, /ga/, /ma/, /fa/, and /va/) required wider DSIRs in the HF ear than DSIRs for other nine consonants. It should also be noted that DSIRs for the two consonants (/ka/ and /ma/) slightly narrowed, compared to those with the 250 Hz. Table 4 shows the results of pairwise multiple comparisons.

**Table 3:** Pairwise multiple comparisons among consonants for the LPF cutoff frequency of 250 Hz.

	ka	ga	ma	fa	va
pa				*	
ba			***	***	***
ta	*	***	***	***	***
da			***	***	***
na				*	
sa	*	*	***	***	***
za	*	**	***	***	***
ʃa			***	***	***
ʒa				**	

^***^p < 0.001; ^**^p < 0.01; ^*^p < 0.05. Consonant pairs with significant difference are only listed.

**Table 4:** Pairwise multiple comparisons among consonants for LPF cutoff frequency of 500 Hz.

	ka	ga	ma	fa	va
pa		***		***	***
ba		***		***	***
ta	*		***	***	***
da		***		***	***
ma				***	***
na		***		***	***
sa	**	***		***	***
za	**	***		***	***
ʃa		***		***	***
ʒa		**		***	***

^***^indicates *p* < 0.001; ^**^p < 0.01; ^*^p < 0.05.Consonant pairs with significant difference are only listed.

With the LPF cutoff frequency of 750 Hz, each consonant required significantly different DSIRs, *F*(13,338) = 6.28, *p* < 0.001, but AI-Gram signal processing did not affect DSIRs significantly, *F*(2,338) = 1.80, *p* = 0.19. There were significant interactions between the variables, *F*(26, 338) = 2.64, *p* < 0.001. With the LPF cutoff frequency of 1,000 Hz, a significant main effect of the consonant was observed, *F*(13,338) = 5.60, *p* < 0.001, but no significant main effect of the AI-Gram signal processing was observed, *F*(2,338) = 1.35, *p* = 0.28. Significant interactions occurred between the type of consonant and AI-Gram signal processing, *F*(26, 338) = 2.95, *p* < 0.001. The patterns of DSIRs are similar between the 750 Hz and 1,000 Hz cutoff frequencies, as observed in the 250 and 500 Hz LPF cutoff frequency conditions, four consonants (/ka/, /ga/, /fa/, and /va/) still required relatively wider DSIRs in the two higher cutoff frequencies. The two consonants, (/fa/ and /va/) in particular, required wider DSIRs than the other two consonants (/ka/ and /ga/). However, /ma/ then had very narrow DSIRs for LFP of 750 and 1,000 Hz for all signal processing conditions. The pairwise multiple comparisons supported these findings. Tables 5, 6 present all pairwise multiple comparisons for the 750 Hz and 1,000 Hz cutoff frequencies, respectively.

**Table 5:** Pairwise multiple comparisons among consonants for LPF cutoff frequency of 750 Hz.

	ka	ga	ma	fa	va
pa	*			***	***
ba	*			**	**
ta	*		**	**	**
da	**			***	***
ma				**	**
sa	**			***	***
za	**			***	***
ʒa				*	*

***p < 0.001; **p < 0.01; *p < 0.05. Consonant pairs with significant difference are only listed.

**Table 6:** Pairwise multiple comparisons among consonants for LPF cutoff frequency of 1,000 Hz.

	ka	ga	ma	fa	va
pa					***
ba					***
ta					***
da				*	***
ka					***
ga					**
ma				***	*
sa				*	***
za				***	*
ʃa					**
ʒa					***

^***^p < 0.001; ^**^p < 0.01; ^*^p < 0.05. Consonant pairs with significant difference are only listed.

### Effect of low-frequency information on DSIRs

Figure 3 presents the actual frequency values of DSIRs per consonant for each low-frequency information available in the LF ear. However, it is hard to remember these frequency values and to see the effect of the different low-frequency information on the DSIR metrics. To generate easier metrics, the DSIRs were converted into percentages of the frequency ranges from the 0–8,000 Hz band. As discussed above in the *Mean DSIR* part of the Results section, these percentages of the DSIRs indicate the upper portion of the 0–8,000 Hz band required for consonant recognition. For example, 70% means 70% of the upper portion of the 0–8,000 Hz band, that is, the 2,400–8,000 Hz range. Figure 4 shows the mean percentages of the DSIRs in the HF ear as a function of the LPF cutoff frequency used in the LF ear.

For the unprocessed condition, four consonants (/ta/, /da/, /sa/, and /za/) required less than 50% of the 0–8,000 Hz band, while two consonants (/fa/ and /va/) needed more than 50% regardless of the LPF cutoff frequency. For the remaining nine consonants, the percentage of the DSIRs varied (more than 20% differences), depending on LPF cutoff frequencies. A two-way repeated measure of ANOVA showed significant effects of the LPF cutoff frequency, *F*(3,507) = 29.64, *p* < 0.001 and of the consonant, *F*(13,507) = 12.85, *p* < 0.001. Significant interactions were also observed, *F*(39,507) = 4.97, *p* < 0.001. Pairwise multiple comparisons with a Bonferroni correction were also performed. However, to demonstrate the different overall effects of the LPF cutoff frequency, the mean differences among the LPF cutoff frequencies were reported rather than to present all pairwise multiple comparisons. The analyses showed significant mean differences between any pairs of the LPF cutoff frequencies (*p* < 0.001) except for the pair 750 vs. 1,000 Hz (*p* = 1.00).

Compared to the unprocessed condition, smaller percentages of DSIRs were needed with the target condition. Seven consonants including the three observed in the unprocessed condition (/ba/, /ta/, /da/, /sa/, /za/, /ʃa/, and /ʒa/) needed less than 50% of the 0–8,000 Hz band regardless of the LPF cutoff frequency, while only /va/ needed more than 50%. The remaining six consonants, including the six observed in the unprocessed condition, exhibited more than 20% differences across the LPF cutoff frequencies. There was a significant difference in the percentage of DSIRs across consonants, *F*(13,507) = 18.52, *p* < 0.001 and the LPF cutoff frequency, *F*(3,507) = 27.41, *p* < 0.001. Significant interactions were also observed, *F*(39,507) = 3.37, *p* < 0.001. Significant mean differences were evident in multiple comparisons between any pairs of the low frequencies (*p* < 0.001), except for the pair 250 vs. 500 Hz (*p* = 0.12) and the pair 750 vs. 1,000 Hz (*p* = 1.00).

For the target-conflicting condition, six consonants (/ba/, /ta/, /da/, /sa/, /za/, and /ʃa/) required less than 50%; however, consonant /fa/ needed more than 50% regardless of the LPF cutoff frequency. The remaining seven consonants, including the five observed in the unprocessed and target conditions, exhibited more than 20% differences across the LPF cutoff frequencies. There was a significant difference in the percentage of DSIRs across consonants, *F*(13,507) = 15.00, *p* < 0.001 and across the LPF cutoff frequency, *F*(3,507) = 18.66, *p* < 0.001. Significant interactions were also observed, *F*(39,507) = 3.82, *p* < 0.001. Multiple comparisons showed significant differences between any pairs of the LPF cutoff frequencies (*p* < 0.001), except for pair 250 vs. 500 Hz (*p* = 0.12) and pair 750 vs. 1,000 Hz (*p* = 1.00).

### Interrelationship among percentages of DSIRs

To quantify the relationship between the changes of DSIRs and different LPF cutoff frequencies, Pearson’s correlation analyses were conducted. Figure 5 shows scatter plots with *r* values and regression lines. As a reference, the DSIRs assessed with the LPF cutoff frequency of 250 Hz were on the x-axis and DSIRs assessed with the other three cutoff frequencies were on the y-axis. Since the DSIR data assessed with the 250 Hz cutoff frequency was used three times for the analyses, a Bonferroni corrected *p* value (i.e., 0.05/3 = 0.017) was used. The overall trends of the analyses show that consonants requiring wide DSIRs in the 250 Hz condition also required wide DSIRs in the 500 Hz condition (and vice versa), but less so in the 750 Hz and 1 kHz conditions. This is consistent across the different AI-gram signal processing. For the unprocessed condition, DSIRs assessed with 250 Hz and 500 Hz (open circles) were significantly correlated, *r*(14) = 0.83, *p* = 0.0002. However, correlation was not significant between 250 Hz and 750 Hz (filled triangles), *r*(14) = 0.61, *p* = 0.021 and between 250 Hz and 1,000 Hz (open diamonds), *r*(14) = 0.49, *p* = 0.08. For the target condition, all three correlations were significant, and *r* values were higher than the corresponding *r* values for the unprocessed condition. The DSIRs between 250 and 500 Hz were strongly correlated, *r*(14) = 0.85, *p* = 0.0001. Correlations were also significant between 250 and 750 Hz, *r*(14) = 0.72, *p* = 0.003 and between 250 and 1,000 Hz, *r*(14) = 0.69, *p* = 0.005. For the target-conflicting condition, all three *r* values were lower than those in the target condition but higher than those in the unprocessed condition. Significant correlations were observed between 250 and 500 Hz, *r*(14) = 0.84, *p* = 0.0001 and between 250 and 750 Hz, *r*(14) = 0.69, *p* = 0.006. However, no significant correlation was observed between 250 and 1,000 Hz, *r*(14) = 0.53, *p* = 0.05.

## Discussion

In this study, frequency ranges needed for consonant recognition in the HF ear were measured when different low-frequency information was simultaneously presented to the LF ear under three signal processing conditions: unprocessed, target, and target-conflicting. The results showed that spectral integration and consonant recognition is possible without midfrequency consonant information. DSIRs were not significantly affected by the two signal processing conditions, except for at the LPF cutoff frequency of 250 Hz in the LF ear. DSIR narrowed significantly with increasing LPF cutoff frequency. Individual consonant analyses showed that four consonants (/ta/, /da/, /sa/, and /za/) required the least amount of spectral information. On the other hand, the four consonants (/ka/, /ga/, /fa/ and /va/) required the widest amount of spectral information. The trends for these nine consonants were consistent, regardless of signal processing and the amount of low-frequency information available in the LF ear. The recognition of the remaining six consonants (/pa/, /ba/, /ma/, /na/, /ʃa/, and /ʒa/) was highly affected by the low-frequency information available in the LF ear regardless of the signal processing condition.

Our finding that consonant recognition is possible without the full range of spectral information is consistent with existing literature. Lippmann (1996) measured consonant-vowel-consonant syllable recognition in quiet with NH listeners when low-frequency information below 800 Hz was combined with high-frequency information above 4,000 Hz in the same ear. The results showed no significant change in consonant recognition when removing midfrequency consonant information (800–4,000 Hz).

It is not surprising that DSIRs were highly consonant specific, regardless of which signal processing condition was used. Four consonants (/ka/, /ga/, /fa/, and /va/), required the widest amount of spectral information regardless of signal processing and the low-frequency information available in the LF ear. It is known that perception of /fa/ and /va/ requires multiple target frequency regions over wide range of spectrum (Allen, 2005). For a pair /ka/ and /ga/, considerable confusions occurred due to same manner and place of articulation (Miller and Nicely, 1955; Allen, 2005), which results in integration with little salient spectral information (Stevens and Klatt, 1974; Stevens and Blumstein, 1978; Stevens et al., 1992). In contrast, four consonants (/ta/, /da/, /sa/, and /za/) required the least amount of spectral information. Perception of these consonants was easier because major spectral cues for their perception were available at 7,000 Hz and beyond (Li et al., 2010, 2012; Li and Allen, 2011; Yoon, 2021). In this study, Sennheiser HAD-200 circumaural headphones were used, which are optimally calibrated with tones but less optimal with speech stimuli. They show a frequency drop-off of about 10 dB for high frequencies compared to low frequencies and hence need to be (free-field or diffuse-field) equalized (ISO389-8, 2004), which was not done in this study. If done appropriately, SDIRs for these four consonants may be further narrowed because their target frequency regions are extended to around 8 kHz.

Our results are similar to the results reported in Lippmann (1996). In that study, six consonants (/p/, /b/, /t/, /k/, /s/, and /z/) were well perceived when combined frequency information lower than 800 Hz and higher than 6,300 Hz was presented simultaneously to one ear (Lippmann, 1996). In contrast, four consonants (/d/, /g/, /f/, and /v/) required combined frequency information lower than 800 Hz and higher than 3,150 Hz. Comparing to our results, recognition of /ka/ required less spectral information. Recognition of /da/ required more spectral information. These differences may stem from different testing paradigms: monotic in the Lippmann study vs. dichotic spectral integration in the current study. Ronan et al. (2004) showed that consonant recognition performance was significantly higher in monotic spectral integration than in dichotic spectral integration in listeners with normal hearing. Spehar et al. (2008) also showed that word recognition was approximately 10 percentage points higher (statistically significant) in monotic spectral integration than dichotic spectral integration for young and elderly listeners with normal hearing. Another reason for different DSIRs, for /da/ and /ka/, between two studies would be the use of different contexts of stimuli: consonant-vowel-consonant vs. consonant-vowel syllables. It is well documented that frequency-time regions that support the robust perception that a consonant is changed if different vowels with different positions of consonants (initial, medial, or final) are used as stimuli (Baum and Blumstein, 1987; Hazan and Rosen, 1991; Reidy et al., 2017).

It should be noted that DSIRs for /fa/ and /va/ were negatively affected by the two signal processing conditions. For /fa/, the widest DSIR was required in the target-conflicting condition and then in the unprocessed and target conditions. Our subject response pattern analysis showed that /ma/ was mostly selected in the target-conflicting condition. This result indicates that removing a conflicting frequency range (3–7.8 kHz) for /fa/ causes confusion with /ma/, requiring the widest DSIR. For /va/, the widest DSIR was required in the target condition and then in the unprocessed and target-conflicting conditions. The subject response patterns showed that /fa/ was mostly selected in the target condition. This result indicates that intensifying a target frequency range (0.6–1.4 kHz) for /va/ causes more confusion with /fa/, requiring the widest DSIR even though target time ranges differ.

Another major finding from the current study was that there was no significant effect of both the AI-Gram processed target and target-conflicting regions on DSIR measures except for the case of the 250 Hz cutoff frequency. However, these processed conditions made spectral cues more prominent and DSIRs were numerically narrower (again not statistically significant) for consonant recognition compared to the unprocessed condition. For example, our analyses (Figure 3) revealed that five consonants (/ta/, /da/, /ka/, /va/, and /za/) for the 250 Hz and another five consonants (/pa/, /ta/, /ka/, /na/, and /va/) for the 500 Hz had narrower DSIRs with two signal-processing conditions than those with the unprocessed condition. This trend was also observed for /pa/, /da/, /fa/, /va/, /za/, and /ʒa/ with the 750 Hz and /pa/, /fa/, and /va/ for the 1,000 Hz.

Our correlation analyses (Figure 5) showed that the DSIRs between 250 Hz and 500 Hz were significantly correlated in all three signal-processing conditions. The correlation was strengthened with the two AI-Gram processed conditions except for the target-conflicting condition between 250 and 1,000 Hz. Similar studies for nonsense phoneme perception were not available, but Hall and colleagues compared sentence perception in NH listeners and reported indirect evidence of this relationship (Hall et al., 2008). They first determined the necessary bandwidth for approximately 15–25% correct scores on sentence perception per listener in both quiet and noise listening environments (called criterion speech bandwidths) by adaptively varying the bandwidth of filtered sentences centered either on 500 Hz or 2,500 Hz. This criterion speech bandwidth measure was conducted monaurally. They found no obvious relation between the criterion bandwidths at each center frequency in both quiet and noise: listeners requiring a relatively wide criterion bandwidth at 500 Hz did not necessarily require a wide bandwidth at 2,500 Hz. This result is not surprising as speech information is widely spread out over a wide range of spectral bands, and the importance of each of these spectral bands for speech perception varies. As Hall et al.’s study (Hall et al., 2008) testing settings were different from ours (e.g., monotic and dichotic), any direct comparisons cannot be made. Our results confirm that the normal auditory system integrates lower spectral information, processed by one ear, with different spectral information processed by the opposite ear.

### Clinical implication

The dichotic test setting of the present study with different low-frequency information in the LF ear could be translated into the four different degrees of high-frequency hearing loss in one ear. The approach may be applied to bimodal users who have residual hearing in low-frequency regions (typically below 1,000 Hz) in the hearing aid ear and can have access to wider frequency information through a cochlear implant in the opposite ear (Gifford et al., 2007; Yoon et al., 2012). So, dichotic spectral integration may play an important role. It is expected that some bimodal listeners with limited access to low-frequency information *via* the hearing aid ear require a broader range of spectral information in the cochlear implant ear. The opposite can occur as well. As shown in Figures 3, 4, DSIRs for six consonants (/pa/, /ba/, /ma/, /na/, /ʃa/, and /ʒa/) were highly sensitive to low-frequency information available in the opposite ear. However, perception of four consonants (/ta/, /da/, /sa/, and /za/) required the narrowest DSIRs, while another four consonants (/ka/, /ga/, /fa/, and /va/) were needed the widest DSIRs, regardless of the signal processing. These results suggest that low-frequency sensitive consonants are most affected by interactions of acoustic and electric stimulations. In bimodal hearing, determining the minimum spectral information needed in a cochlear implant ear for consonant-by-consonant perception on an individual, subject-by-subject basis is critical because interactions across ears are highly listener specific (Cullington and Zeng, 2010; Gifford and Dorman, 2019; Shpak et al., 2020). Fowler et al. (2016) measured speech perception with bimodal listeners as a function of high-pass cutoff frequency for the cochlear implant ear. Speech perception with the cochlear implant ear alone deteriorated as the high-pass cutoff frequency was raised. In contrast, bimodal performance in quiet was improved as the high-pass cutoff frequency was raised for listeners with better residual hearing in a hearing aid ear (< 60 dB HL at 250 and 500 Hz). This result suggests that determining minimum spectral information needed in a cochlear implant ear can reduce spectral interference in bimodal hearing (Fowler et al., 2016). Consonant-specific and listener-specific datasets are also necessary to train a neural network-based deep machine learning algorithm which is currently in progress in our laboratory. Training the deep machine learning algorithm will be effective with our consonant-by-consonant datasets for maximizing algorithm accuracy and minimizing errors (Vaerenberg et al., 2011; Wang, 2017; Wathour et al., 2020). Hence, the present study findings will aid in designing custom bimodal frequency maps for greater consonant intelligibility based on residual hearing available in the hearing aid ear. One caution of direct application into bimodal hearing is that simulating a hearing aid ear requires careful incorporating gains with specific input levels for each band on a patient-by-patient basis using clinical prescription procedures (Zhang et al., 2010; Sheffield et al., 2016), which were not done in the current study.

Currently, our laboratory has conducted a series of bimodal simulation studies to derive the frequency importance function of cochlear implant ear and combined cochlear implant and hearing aid ears. In addition, a spectral integration and interference study is ongoing for vowel and consonant recognition with manipulation of first and second formant frequencies. The present datasets will serve as a control for some ongoing studies. Our long-term goal of the AI-Gram based speech recognition studies is to develop efficient bimodal fitting schemes based on deep machine learning. It is expected that the target and conflicting frequency and time regions, reported in Yoon (2021), in conjunction with the expected results of the bimodal study, the minimum spectral information required for consonant recognition in cochlear implant ears would be effective in training algorithms.

### Limitations

The present study has several limitations. First, using a single female talker creates a clear limitation of talker variability in real listening situations. The target and conflicting regions might differ depending on different talkers (Mullennix et al., 1989; Goldinger et al., 1991; Magnuson and Nusbaum, 2007). Thus, DSIR may also vary widely across talkers, particularly for listeners with hearing loss and hearing devices. However, based on comparable data in the target and conflicting regions between the current study and Li et al. (2010, 2012), different talkers may affect these regions less substantially. Second, the baseline performance for each ear alone was not measured. Our data was likely a result of the dichotic spectral integration. However, it is possible that consonant recognition could be achieved with higher frequency spectral information only, particularly for some consonants such as /sa/ and /ʃa/. Third, the single phonetic environment (consonant+/a/ vowel) was used. Critical spectral-temporal regions that facilitate or limit our ability to integrate auditory information might change if different consonant-vowel combinations are used at different positions (initial, medial, or final) as stimuli (Hayden, 1950; Harris, 1958; Soli, 1981; Viswanathan et al., 2010). Finally, one technical concern is the possibility that optimal spectral integration may occur with different suppression levels to completely remove conflicting frequency and time regions used in the current study. In our pilot study with five NH listeners, a wide range of suppression from −2 to −20 dB in every 2 dB decrement were tested. No additional consonant enhancement was seen with higher than −6 dB for fricative consonants and less than 2% consonant enhancement for stop consonants. With the complete removal of the conflicting regions, speech perception was significantly enhanced for all consonants except /sa/ and /ʃa/, whose perception suffered by 15% compared to the unprocessed condition. Hence, though not studied in the present study, the removal of conflicting frequency and time regions alone as a condition may be studied vastly in future works.

## Data availability statement

The raw data supporting the conclusions of this article will be made available by the authors, without undue reservation.

## Ethics statement

The studies involving human participants were reviewed and approved by Baylor University (IRB ID: #125371). The patients/participants provided their written informed consent to participate in this study.

## Author contributions

Y-SY conceived and designed the study, analyzed the data, and wrote the draft of the manuscript. DM collected the data. All authors contributed to the article and approved the submitted version.

## Funding

This work was supported by the National Institutes of Health under R15DC019240. The funder was not involved in the study design, collection, analysis, interpretation of data, the writing of this article, or the decision to submit it for publication.

## Conflict of interest

The authors declare that the research was conducted in the absence of any commercial or financial relationships that could be construed as a potential conflict of interest. The handling editor declared a past collaboration with one of the authors, Y-SY.

## Publisher’s note

All claims expressed in this article are solely those of the authors and do not necessarily represent those of their affiliated organizations, or those of the publisher, the editors and the reviewers. Any product that may be evaluated in this article, or claim that may be made by its manufacturer, is not guaranteed or endorsed by the publisher.
